# SGLT-2 Inhibitors and GLP-1 Receptor Agonists as Combination Therapy in Type 2 Diabetes

**DOI:** 10.1007/s11892-025-01616-z

**Published:** 2026-01-13

**Authors:** Aris Liakos, Thomas Karagiannis, Ioannis Avgerinos, Eleni Bekiari

**Affiliations:** 1 Clinical Research and Evidence-Based Medicine Unit, Second Medical Department, Aristotle University of Thessaloniki, Thessaloniki, Greece; 2 Diabetes Centre, Second Medical Department, Aristotle University of Thessaloniki, Thessaloniki, Greece

**Keywords:** SGLT-2 inhibitors, GLP-1 receptor agonists, Combination therapy, Type 2 diabetes

## Abstract

**Purpose of Review:**

The pharmacologic management of type 2 diabetes prioritises sodium-glucose cotransporter 2 (SGLT-2) inhibitors or glucagon-like peptide 1 (GLP-1) receptor agonists for their demonstrated cardiovascular benefits in individuals with atherosclerotic cardiovascular disease or multiple cardiovascular risk factors, chronic kidney disease, and heart failure. However, while current guidelines recommend these drug classes alone, combination therapy is not explicitly advocated. Herein we summarise the rationale and available evidence in support for combination therapy.

**Recent Findings:**

Evidence suggests that combining SGLT-2 inhibitors and GLP-1 receptor agonists improves metabolic outcomes, including HbA_1c_, body weight, and blood pressure. More importantly, combination therapy can offer potential advantages for addressing residual cardiovascular risk, particularly in high-risk populations. Data from cardiovascular outcomes trials and real-world studies demonstrate consistent benefits of combination therapy across diverse subpopulations, including those with established atherosclerotic cardiovascular disease or chronic kidney disease. However, robust evidence remains limited for individuals at low cardiovascular risk, where therapy should primarily focus on metabolic goals. Of note, combination therapy faces significant barriers, including safety concerns in older or frail individuals, underutilisation in disadvantaged populations, while economic challenges may further hinder the accessibility of these therapies.

**Summary:**

Upfront combination therapy with both SGLT-2 inhibitors and GLP-1 receptor agonists could further reduce cardiovascular risk in people with type 2 diabetes, although it is crucial to pare down cost and disparities to access to maximise widespread benefits at population level.

## Introduction

Clinical practice recommendations for the pharmacologic management of type 2 diabetes prioritise sodium-glucose cotransporter 2 (SGLT-2) inhibitors for people with comorbid heart failure (HF) or chronic kidney disease (CKD) and either glucagon-like peptide 1 (GLP-1) receptor agonists or SGLT-2 inhibitors for those with atherosclerotic cardiovascular disease (CVD) or with multiple cardiovascular risk factors [1, 2]. Despite advances in cardiovascular risk mitigation with each one of those drug classes, treatment algorithms do not explicitly advocate combination regimens with both SGLT-2 inhibitors and GLP-1 receptor agonists, potentially leaving a substantial amount of cardiovascular risk unaddressed especially for individuals with manifest cardiorenal disease. Concomitant therapy may be already occurring naturally in everyday clinical practice, but it remains questionable whether it truly maximises cardiorenal benefits or if it could just represent a wasteful use of resources. In this work we review current evidence and argue that combination therapy should be primarily deployed for high-risk patients to maximise cardiorenal benefits, provided cost and access barriers are addressed.

### Rationale for Combination Therapy

SGLT-2 inhibitors induce natriuresis and osmotic diuresis which in turn decrease cardiac preload and afterload, alleviate increased intraglomerular pressure and exert a downstream effect on blood pressure. In addition to improving systemic and renal haemodynamics, they stimulate erythropoiesis leading to improved oxygenation of ischemic tissues and shift cardiac metabolic substrate utilization from glucose to ketones. GLP-1 receptor agonists delay gastric emptying, increase satiety and have several pleiotropic actions such as improvements in low grade inflammation and endothelial dysfunction thereby stabilising atherosclerotic plaques [3]. Both drug classes lower glucose concentrations (though through distinct mechanisms) without excess risk for hypoglycaemia, reduce body weight and albuminuria and prevent cardiovascular events, but they have opposing effects on glucagon secretion which is augmented by SGLT-2 inhibitors but suppressed by GLP-1 receptor agonists. In fact, GLP-1 receptor agonists have a more favourable profile in terms of glycaemic control and body weight reduction, whereas in terms of hard clinical endpoints SGLT-2 inhibitors predominantly reduce hospitalisations for worsening HF and ameliorate progression of CKD, while GLP-1 receptor agonists may have more pronounced effects against myocardial infarction and ischaemic stroke [4].

### Effect of Combination Therapy on Metabolic Outcomes

Based on a 2019 meta-analysis of seven randomised controlled trials (1,913 participants) with a primary duration ranging from 12 to 30 weeks, co-administration of SGLT-2 inhibitors and GLP-1 receptor agonists had a less than additive effect on HbA_1c_ and body weight and an additive reduction in systolic blood pressure compared with each monocomponent without increasing risk of hypoglycaemia [5]. Similarly, another meta-analysis incorporating data until 2021 concluded that combination therapy has superior efficacy for controlling fasting and post-prandial hyperglycaemia and reducing low-density lipoprotein cholesterol (LDL-C) levels. Adverse events leading to discontinuation including hypoglycaemia, vomiting, and diarrhoea were more common with combination regimens [6]. A more recent meta-analysis including studies published up to 2023 showed that the aforementioned metabolic benefits of combination therapy do not likely exceed those of GLP-1 receptor agonists alone [7].

Despite these findings small mechanistic studies evaluating the metabolic and vascular effects of combination treatment have been ambiguous. Regarding kidney function, the DECREASE trial which enrolled 66 participants with obesity and type 2 diabetes showed a mean percentage change in 24-h urine albumin to creatinine ratio (UACR) of 40% with combination treatment including dapagliflozin and exenatide as opposed to 18% with dapagliflozin, 16% with exenatide and 11% with placebo [8], although between group differences did not reach statistical significance. Numerically greater reduction in albuminuria amongst patients receiving dapagliflozin plus exenatide was also observed in another 6-week cross-over study (DECADE) that recruited 20 participants with type 2 diabetes [9]. Adding semaglutide on top of empagliflozin did not also improve UACR in individuals with type 2 diabetes and albuminuria [10]. In terms of vascular function, combination therapy with empagliflozin and semaglutide did not improve arterial stiffness as assessed by means of carotid-femoral pulse wave velocity neither kidney oxygenation and perfusion evaluated with magnetic resonance imaging [11, 12]. Finally, in a small trial utilising magnetic resonance spectroscopy (EXENDA) no significant changes were evident in hepatocellular lipids or visceral adiposity between patients receiving combination treatment with exenatide plus dapagliflozin and those treated with dapagliflozin alone [13].

### Effect of Combination Therapy on Residual Cardiovascular Risk

Residual cardiovascular risk refers to the cardiovascular events that occur despite appropriate guideline-directed medical therapy, a concept first introduced for lipid-lowering therapies [14]. Even among individuals treated with both statins and proprotein convertase subtilisin kexin type 9 inhibitors, a major adverse cardiovascular event (MACE) rate of approximately 7% per year has been observed, indicating that substantial cardiovascular risk remains, even with optimal LDL-C reduction [15]. This persistent risk has been attributed to additional factors, such as triglyceride-rich lipoproteins, remnant cholesterol, lipoprotein(a), and chronic inflammation.

**Fig. 1:**
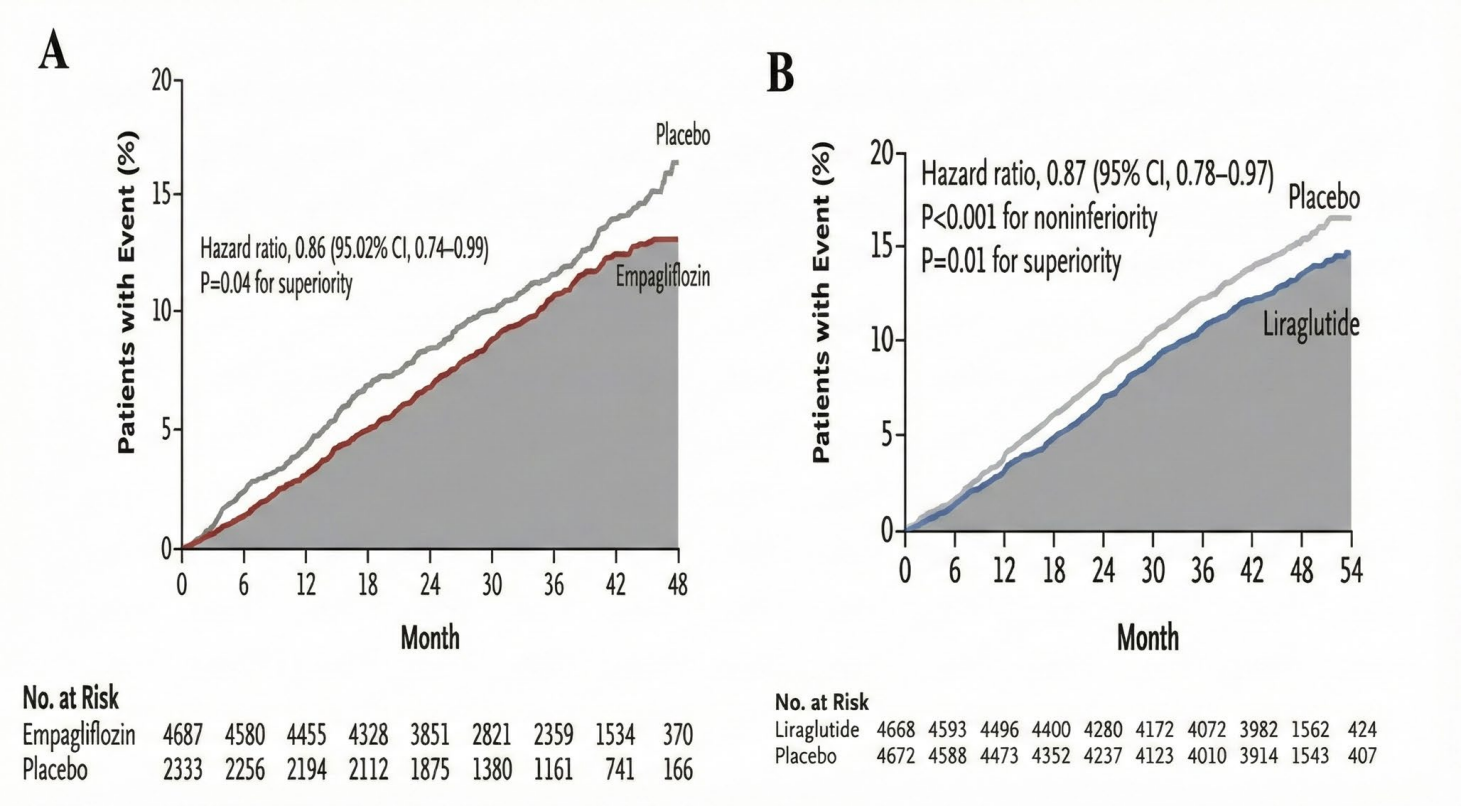
Incidence of major adverse cardiovascular events amongst people with type 2 diabetes in the (**A**) EMPA-REG and (**B**) LEADER trials with empagliflozin and liraglutide respectively. Grey shaded areas represent residual cardiovascular risk. All subjects enrolled in the EMPAREG-OUTCOME trial had established cardiovascular disease, whereas participants in the LEADER trial had either previous cardiovascular disease or multiple risk factors. Although the level of underlying risk differs, a substantial amount of residual risk remains unaddressed with isolated use of sodium-glucose cotransporter 2 inhibitors or glucagon-like peptide 1 receptor agonists. From Zinman B, Wanner C, Lachin JM, Fitchett D, Bluhmki E, Hantel S, et al. Empagliflozin, cardiovascular outcomes, and mortality in type 2 diabetes. N Engl J Med. 2015;373:2117–28 and Marso SP, Daniels GH, Brown-Frandsen K, Kristensen P, Mann JFE, Nauck MA, et al. Liraglutide and cardiovascular outcomes in type 2 diabetes. N Engl J Med. 2016;375:311–22. Copyright ^©^ 2015-16 Massachusetts Medical Society. Reprinted with permission from Massachusetts Medical Society

In type 2 diabetes, residual cardiovascular risk is evident in individual cardiovascular outcome trials (CVOTs) of SGLT-2 inhibitors and GLP-1 receptor agonists. For example, in the EMPA-REG OUTCOME trial, while empagliflozin significantly reduced the hazard ratio (HR) for MACE, 10.5% of participants treated with empagliflozin still experienced a MACE event, compared to 12.1% in the placebo arm [16]. Similarly, in the LEADER trial, MACE occurred in 13.0% of participants receiving liraglutide, compared to 14.9% in the placebo arm [17] (Fig. 1). In a meta-analysis of CVOTs, the 5-year absolute risk reduction (ARR) in MACE with SGLT-2 inhibitors compared to placebo was 1.8% in people with established atherosclerotic CVD, and 0.8% in those with multiple cardiovascular risk factors only [18]. For GLP-1 receptor agonists, the 5-year ARR was 3.0% in patients with established atherosclerotic CVD, and 1.4% in those with multiple cardiovascular risk factors only [18]. These findings suggest that a substantial residual cardiovascular risk remains even after treatment with either a SGLT-2 inhibitor or a GLP-1 receptor agonist. Specifically, in people with established atherosclerotic CVD, where the 5-year baseline cardiovascular risk was 22.3%, treatment with SGLT-2 inhibitors alone leaves a residual risk of 20.5%, while treatment with a GLP-1 receptor agonist alone leaves a residual risk of 19.3%. In people with cardiovascular risk factors but without manifest CVD, where the baseline risk was 9.4%, the residual risk after treatment remains at 8.6% with SGLT-2 inhibitors and 8.0% with GLP-1 receptor agonists (Table 1) [18].

**Table 1:** Five-year anticipated absolute risk reduction for major adverse cardiovascular events comparing treatment with sodium-glucose cotransporter 2 inhibitors or glucagon-like peptide 1 receptor agonists versus placebo in people with type 2 diabetes. Data are based on work by Karagiannis et al. [18]

Comparison	Subgroup population	Five-year absolute risk reduction (95% CI)	Residual cardiovascular risk
SGLT2 inhibitors vs. placebo	Established cardiovascular disease	1.8% (0.6 to 3.0)	20.5%
SGLT2 inhibitors vs. placebo	Cardiovascular risk factors or subclinical cardiovascular disease	0.8% (0.3 to 1.4)	8.6%
GLP-1 receptor agonists vs. placebo	Established cardiovascular disease	3.0% (2.0 to 4.0)	19.3%
GLP-1 receptor agonists vs. placebo	Cardiovascular risk factors or subclinical cardiovascular disease	1.4% (0.9 to 1.8)	8.0%

*CI* confidence interval, *MACE* major adverse cardiovascular events (cardiovascular death or myocardial infarction or stroke)

#### People with Established or with Multiple Risk Factors for Atherosclerotic CVD

The impact of combining SGLT-2 inhibitors and GLP-1 receptor agonists on cardiorenal outcomes has been evaluated in subgroup meta-analyses of CVOTs involving populations with type 2 diabetes and either established atherosclerotic CVD or multiple cardiovascular risk factors only. A meta-analysis involving more than 70,000 participants with type 2 diabetes (SMART-C) found that the cardiovascular benefits of SGLT-2 inhibitors, including reductions in MACE, cardiovascular death, hospitalisation for HF, and CKD progression, were consistent regardless of whether patients were receiving a GLP-1 receptor agonist at baseline [19]. Similarly, a subgroup meta-analysis of CVOTs with GLP-1 receptor agonists reported that the reduction in MACE was consistent regardless of whether participants were already being treated with an SGLT-2 inhibitor at baseline [20].

#### People with Chronic Kidney Disease

Evidence regarding the effect of combination treatment with GLP-1 receptor agonists and SGLT-2 inhibitors in people with type 2 diabetes and CKD comes from a cross-trial simulation analysis of cardiovascular and renal outcome trials. Based on this simulation combination therapy reduced HRs for several cardiorenal endpoints including incidence of MACE, HF hospitalisation and CKD progression compared to placebo more effectively than either drug class alone [21].

Of note, the evidence for people with type 2 diabetes and CKD is more robust for SGLT-2 inhibitors, given that dedicated cardiorenal outcome trials have consistently demonstrated significant cardiorenal protection in this subpopulation. In contrast, relevant data for GLP-1 receptor agonists are limited to the recently published FLOW trial, which demonstrated substantial cardiorenal benefits with subcutaneous semaglutide in people with type 2 diabetes and CKD [22]. Notably, the benefits of semaglutide in kidney outcomes were consistent irrespective of SGLT-2 inhibitor use at baseline [23].

#### People with Heart Failure

High quality evidence for the combination of SGLT-2 inhibitors and GLP-1 receptor agonists in people with type 2 diabetes and HF is scarce. SGLT-2 inhibitors have well-established benefits in individuals with HF, regardless of diabetes status, as demonstrated in several long-term, dedicated HF trials across a wide spectrum of HF patients, including those with reduced and preserved ejection fraction [24]. Improvements in heart failure outcomes with GLP-1 receptor agonists have thus far been demonstrated only with specific agents such as subcutaneous semaglutide [25], the first in class dual glucose-dependent insulinotropic polypeptide/GLP-1 receptor agonist tirzepatide [26], as well as the nonsteroidal mineralocorticoid receptor antagonist finerenone [27]. Nevertheless, the HF benefits for those drug classes beyond those conferred by SGLT-2 inhibitors have been only ascertained in patients with HF with preserved or mildly reduced ejection fraction.

#### People at Low Cardiovascular Risk

In people with type 2 diabetes at low cardiovascular risk—those without atherosclerotic CVD, HF, CKD, or multiple cardiovascular risk factors—long-term data on cardiovascular outcomes from randomised controlled trials are lacking. The GRADE trial provides some insights, reporting a cardiovascular benefit with liraglutide; however, the trial did not specifically target low cardiovascular risk populations, even though participants had a relatively lower baseline cardiovascular risk compared to CVOTs [28]. Moreover, even if the cardiovascular benefits of GLP-1 receptor agonists or SGLT-2 inhibitors extend to low-risk populations, the absolute benefits are attenuated due to the low baseline cardiovascular risk in this population. As such, therapy in low-risk individuals should be guided by treatment effects on metabolic outcomes, such as HbA_1c_ reduction and weight loss, rather than cardiovascular outcomes. Combination therapy with GLP-1 receptor agonists and SGLT-2 inhibitors could be considered for further metabolic improvements, but newer dual incretin therapies, such as tirzepatide, may be more potent in reducing HbA_1c_ and body weight [29].

#### Observational Data from Routine Care

Additional evidence regarding the effectiveness of combination therapy is available from observational studies. In a real-world study from Denmark SGLT-2 inhibitor and GLP-1 receptor agonist combination resulted in lower five year rates of all-cause mortality, HF, end-stage kidney disease, and > 50% decline in eGFR compared to each individual drug class [30, 31]. The comparative benefits of combination therapy have been demonstrated both for primary and secondary prevention. From a retrospective cohort of 122,458 people with type 2 diabetes, established CVD and heart failure in the US, combination therapy reduced the composite of all-cause mortality, myocardial infarction and non-fatal stroke by 67% within one year compared with propensity score matched subjects treated with SGLT-2 inhibitors alone [32]. Based on findings from three nested case-control studies with 336,334 participants conducted in the UK the positive effects on MACE and HF hospitalisations were also sustained amongst individuals without CVD [33]. Recent nationwide data from the UK suggested that combination therapy was associated with lower rates of cardiovascular and serious renal events compared with either drug class alone [34]. Another retrospective cohort study including patients with type 2 diabetes and HF with preserved ejection fraction showed an incremental benefit of combination therapy in reducing HF exacerbations [35].

Taken together, combining an SGLT-2 inhibitor with a GLP-1 receptor agonist in people with type 2 diabetes is associated with lower risk of major cardiovascular events, mortality, heart failure hospitalisations and adverse kidney outcomes compared with monotherapy based on a meta-analysis of 18 cohort studies with 1,164,774 participants [36]. Another meta-analysis synthesising all available data from randomised controlled trials, post-hoc analyses and observational studies showed consistent findings [37]. Going even further, a more recent evidence synthesis found that dual combination therapy with either SGLT-2 inhibitors, GLP-1 receptor agonists or finerenone offers significant cardiovascular and renal protection [38]. PRECIDENTD is an ongoing, pragmatic randomised trial that will directly compare GLP-1 receptor agonists with SGLT-2 inhibitors for CVD and kidney events (NCT05390892). This independently funded, comparative effectiveness trial also included a dual combination arm that was regretfully closed after the pilot phase.

The available evidence from different study types for the effects of combination therapy with SGLT-2 inhibitors and GLP-1 receptor agonists is summarised in Table 2.

**Table 2:** Available evidence for the effects of combination therapy with sodium-glucose cotransporter 2 (SGLT-2) inhibitors and glucagon-like peptide 1 (GLP-1) receptor agonists

Study, acronym	Population	Comparison	Key results
**Mechanistic studies**			
Harreiter et al.[13], EXENDA	30 people with T2D on metformin and BMI ≥ 25 kg/m^2^	Dapagliflozin vs. exenatide once weekly for 24 weeks	Similar hepatocellular lipid reduction assessed with magnetic resonance spectroscopy in both groups
Sivalingam et al. [10]	60 people with T2D and albuminuria	Semaglutide vs. placebo on top of empagliflozin for 26 weeks	No additional change in UACR
van der Aart-van der Beek et al. [9], DECADE	20 people with T2D and albuminuria	Dapagliflozin vs. exenatide once weekly, alone or in combination for 6 weeks	Greater UACR reduction of 26.0% in the combined exenatide-dapagliflozin group
van Ruiten et al. [8], DECREASE	66 people with T2D and obesity	Dapagliflozin and exenatide twice daily, alone or in combination, vs. placebo for 16 weeks	Greater UACR reduction of 39.6% in the combined exenatide-dapagliflozin group
Vernstrøm et al. [11]	120 people with T2D	Empagliflozin and semaglutide once weekly, alone or in combination, vs. placebo for 32 weeks	No significant change in arterial stiffness assessed with carotid-femoral pulse wave velocity or kidney oxygenation assessed with magnetic resonance imaging
**Systematic reviews and meta-analyses for metabolic outcomes**			
Li et al. [6]	8 RCTs with 1,895 people with T2D	Combination of SGLT-2 inhibitors with GLP-1 receptor agonists vs. either drug class alone	Superior efficacy of combination therapy on HbA_1c_, body weight, fasting and post-prandial glucose, systolic blood pressure and low density lipoprotein cholesterol
Mantsiou et al. [5]	7 RCTs with 1,913 people with T2D	Combination of SGLT-2 inhibitors with GLP-1 receptor agonists vs. either drug class alone	Superior efficacy of combination therapy on HbA_1c_, body weight and systolic blood pressure without increased risk of hypoglycaemia
**Meta-analyses of subgroups from cardiorenal outcome trials**			
Apperloo et al. [19], SMART-C	12 RCTs with 73,238 people with T2D	SGLT-2 inhibitors with or without background therapy with GLP-1 receptor agonists	Consistent cardiovascular and kidney benefits of SGLT-2 inhibitors regardless of background therapy with GLP-1 receptor agonists
Neuen et al. [20]	3 RCTs with 1,743 people with T2D	GLP-1 receptor agonists with or without background therapy with SGLT-2 inhibitors	Consistent cardiovascular and kidney benefits of GLP-1 receptor agonists regardless of background therapy with SGLT-2 inhibitors
**Large observational studies**			
Clemmensen et al. [30] Zareini et al. [31]	People with T2D from Denmark starting dual second line therapy	Combination of SGLT-2 inhibitors with GLP-1 receptor agonists vs. dual therapy with either dipeptidyl-peptidase 4 inhibitors, sulphonylureas or thiazolidinediones	Lower risk of all-cause mortality, MACE, end-stage renal disease and estimated glomerular filtration rate decline with combination therapy
Lopez et al. [32]	People with T2D, CVD and HF from the US	Combination of SGLT-2 inhibitors with GLP-1 receptor agonists vs. SGLT-2 inhibitors alone	Lower risk of of all-cause mortality, myocardial infarction and stroke with combination therapy
Patel et al. [35]	People with T2D, BMI ≥ 27 kg/m^2^ and HF with preserved ejection fraction from the US	SGLT-2 inhibitors with or without background therapy with GLP-1 receptor agonists	Lower risk of HF hospitalisations with the addition of GLP-1 receptor agonists on background therapy with SGLT-2 inhibitors
Simms-Williams et al. [34]	People with T2D from the United Kingdom starting either SGLT-2 inhibitors or GLP-1 receptor agonists	Combination of SGLT-2 inhibitors with GLP-1 receptor agonists vs. either drug class alone	Lower risk of MACE and serious renal events with combination therapy
Wright et al. [33]	People with T2D without CVD from the UK	Combination of SGLT-2 inhibitors with GLP-1 receptor agonists vs. either drug class alone	Lower risk of MACE and HF with combination therapy
**Systematic review and meta-analysis of observational studies**			
Colombijn et al. [36]	18 cohort studies with 1,164,774 people with T2D	Combination of SGLT-2 inhibitors with GLP-1 receptor agonists vs. either drug class alone	Lower risk of MACE, all-cause and cardiovascular mortality, hospitalisations for heart failure and kidney composite endpoints

*BMI* body mass index, *CVD* cardiovascular disease, *HbA*_1c_ haemoglobin A_1c_, *HF* heart failure, *MACE* major adverse cardiovascular events, *RCT* randomised controlled trial, *T2D* type 2 diabetes, *UACR* urine albumin to creatinine ratio

### Implications for Practice

Current clinical practice recommendations acknowledge a potential additive cardiovascular and kidney benefit from using both a GLP-1 receptor agonist and a SGLT-2 inhibitor. Notably though, the accompanying treatment algorithms do not explicitly recommend combination therapy. Of note, the ADA supports a multifactorial approach to reducing cardiovascular risk and diabetes-related complications emphasising the importance of managing glycaemic control, blood pressure, and lipid levels, alongside incorporating therapies with proven cardiovascular and kidney benefits. These four pillars align with the concept that reducing cardiovascular risk in people with type 2 diabetes often requires multiple interventions to address various risk factors simultaneously [39].

Herein, we extend this concept further by focusing specifically on the role of combination therapy as a more nuanced approach to mitigate residual cardiovascular risk in people with type 2 diabetes. Specifically, for individuals with established atherosclerotic CVD or comorbid CKD, combination therapy with SGLT-2 inhibitors and GLP-1 receptor agonists could be used to further reduce cardiovascular events. On the contrary, for individuals with comorbid HF or without atherosclerotic CVD, combination therapy may be considered for improving metabolic outcomes given the scarcity of evidence and that any absolute benefits on cardiorenal outcomes are expected to be attenuated. Using both drug classes in combination could be reasonable not just in a sequential manner primarily driven by glycaemic control, but as a simultaneous initiation regardless of HbA_1c_ levels, particularly in people with established or at high risk for atherosclerotic CVD and those with CKD.

Interpretation of the available evidence supporting combination therapy warrants caution. Much of the apparent benefit derives from subgroup analyses of CVOTs and observational studies, the latter being inherently susceptible to treatment selection bias and residual confounding despite statistical adjustment. Patients receiving combination therapy are often younger, have better access to healthcare, and are more likely to be managed by specialists, factors that are incompletely captured in routine datasets and may independently influence outcomes. In addition, heterogeneity in outcome definitions across studies—particularly for composite cardiovascular endpoints, heart failure hospitalisation, and kidney disease progression—limits direct comparability. These methodological shortcomings reduce certainty regarding causality and preclude strong recommendations at this stage, especially for individuals at low cardiovascular risk. Dedicated prospective trials evaluating combination strategies with harmonised outcome definitions are needed.

Looking ahead, there is potential for expanding combination therapy beyond the current dual therapy approach. Data presented for tirzepatide suggest similar cardioprotective effects to other GLP-1 receptor agonists [40]. The contemporary CVOTs for tirzepatide will recruit a larger proportion of patients on background SGLT-2 inhibitor therapy and are hence expected to better clarify the effect of combination therapy. Oral, small molecule, non-peptide GLP-1 receptor agonists such as orforglipron, which has demonstrated high antihyperglycaemic and weight loss potency in the ACHIEVE clinical development programme [7], may ultimately expedite the advent of all-in-one combinations with SGLT-2 inhibitors. Additionally, combination of SGLT-2 inhibitors with finerenone leads to greater reduction of albuminuria [41] and triple therapy with the addition of a GLP-1 receptor agonist may offer even more pronounced cardiovascular risk reductions [21]. Beyond cardiac and kidney benefits, in people with type 2 diabetes and metabolic-associated steatotic liver disease a network meta-analysis indicated that each one of GLP-1 receptor agonists and SGLT-2 inhibitors have a favourable effect on liver steatosis [42], thereby paving the way for evaluating combination regimens for this indication. In the future, a precision medicine approach aiming at identifying clinical and biological characteristics associated with variations in treatment response might enable more personalised decisions when selecting the most appropriate glucose lowering combination for individuals with type 2 diabetes [43].

### Barriers in the Implementation of Combination Therapy

Clinically important adverse events, accessibility, equity, and cost-effectiveness issues should also be taken into account when considering a combination treatment. A meta-analysis of short-term RCTs indicated that adding GLP-1 receptor agonists to SGLT-2 inhibitors increased the risk of hypoglycaemia and treatment discontinuation due to adverse events [6]. SGLT-2 inhibitors could increase the risk of euglycaemic diabetic ketoacidosis (DKA) and genitourinary tract infections as well as acute renal damage and volume depletion, especially when co-administered with diuretics. In frail and older populations these safety concerns could be amplified. A meta-analysis of 39 randomised controlled trials suggested that the incidence of DKA in people treated with SGLT-2 inhibitors rises with age [44]. SGLT-2 inhibitors may adversely affect muscle mass, contributing to an increased risk of sarcopenia in frail individuals. Similarly, the use of GLP-1 receptor agonists in older adults has raised questions regarding lean mass changes, though the data are more heterogeneous. Some clinical trials report that lean mass reductions can account for 40% to 60% of total weight lost, while others show reductions of around 15% or less [45, 46].

Research across various countries and healthcare settings has consistently shown that both SGLT-2 inhibitors and GLP-1 receptor agonists are underused, particularly in socioeconomically disadvantaged populations and minority groups, even though these populations often face a higher CVD burden and could potentially benefit the most from these treatments [47–49]. A significant barrier to access is the high out-of-pocket costs of these medications, particularly for more expensive drugs like newer GLP-1 receptor agonists [49]. The high cost of these newer medications remains a major issue in terms of achieving value for money from a societal perspective. In particular, a price-target analysis covering 67 low- and middle-income countries found that GLP-1 receptor agonists, unlike SGLT-2 inhibitors, were generally not cost-effective in these countries [50]. Moreover, a systematic review of cost-effectiveness in the US concluded that both SGLT-2 inhibitors and GLP-1 receptor agonists offer low value as first-line therapies, although they may provide intermediate value when used as second-line therapies [51]. Considering these challenges and given the paucity of cost-effectiveness data regarding combination therapy, it is reasonable to assume that these socioeconomic barriers may be amplified when both drug classes are used in combination.

## Conclusion

While combination therapy with SGLT-2 inhibitors and GLP-1 receptor agonists holds promise for addressing residual cardiovascular risk in type 2 diabetes, its optimal use requires nuanced consideration of patient subpopulations, safety, and cost-effectiveness. Evidence supports significant benefits in individuals with high cardiorenal risk, but barriers such as adverse effects particularly in older individuals, underutilisation in disadvantaged groups, and economic challenges may limit widespread adoption.

##  Key References

- Efficacy and safety of SGLT2 inhibitors with and without glucagon-like peptide 1 receptor agonists: a SMART-C collaborative meta-analysis of randomised controlled trials. Lancet Diabetes Endocrinol. 2024;12:545–57.
  - **○ **Due to the absence of large randomised trials directly evaluating combined SGLT-2 inhibitor and GLP-1 receptor agonist therapy, this work synthesises data from major cardiorenal outcome trials. It shows that the cardiovascular and kidney benefits of SGLT-2 inhibitors are consistent regardless of background GLP-1 receptor agonist use.
- Cardiovascular, kidney and safety outcomes with GLP-1 receptor agonists alone and in combination with SGLT2 inhibitors in type 2 diabetes: a systematic review and meta-analysis. Circulation. 2024;149:450–62.
  - **○ **This systematic review and meta-analysis assessed GLP-1 receptor agonist therapy with and without SGLT-2 inhibitors, finding comparable reductions in cardiovascular events, heart failure hospitalisations, and kidney disease progression
- Effect of combination treatment with glucagon-like peptide-1 receptor agonists and sodium-glucose cotransporter-2 inhibitors on incidence of cardiovascular and serious renal events: population based cohort study. BMJ. 2024;384:e078242.
  - **○ **A large population-based cohort study showing that combined SGLT-2 inhibitor and GLP-1 receptor agonist use is associated with approximately 30% lower risk of major adverse cardiovascular and serious renal events compared with monotherapy.
- Effectiveness and safety of combining SGLT2 inhibitors and GLP-1 receptor agonists in individuals with type 2 diabetes: a systematic review and meta-analysis of cohort studies. Diabetologia. 2026;69:36–49.
  - **○ **This meta-analysis of 18 cohort studies with more than one million participants concluded that combining an SGLT-2 inhibitor with a GLP-1 receptor agonist is associated with better cardiovascular, heart failure and kidney outcomes.

## Data Availability

No datasets were generated or analysed during the current study.
